# The Expression of Trace Amine-Associated Receptors (TAARs) in Breast Cancer Is Coincident with the Expression of Neuroactive Ligand–Receptor Systems and Depends on Tumor Intrinsic Subtype

**DOI:** 10.3390/biom13091361

**Published:** 2023-09-07

**Authors:** Anastasia N. Vaganova, Daria D. Maslennikova, Valeria V. Konstantinova, Evgeny V. Kanov, Raul R. Gainetdinov

**Affiliations:** 1 Institute of Translational Biomedicine, St. Petersburg State University, Universitetskaya nab. 7/9, 199034 St. Petersburg, Russia; a.n.vaganova@spbu.ru (A.N.V.); e.kanov@spbu.ru (E.V.K.); 2 St. Petersburg University Hospital, St. Petersburg State University, Universitetskaya nab. 7/9, 199034 St. Petersburg, Russia; v.konstantinova@spbu.ru; 3 Faculty of Biology, St. Petersburg State University, Universitetskaya nab. 7/9, 199034 St. Petersburg, Russia; st090193@student.spbu.ru

**Keywords:** trace amines, trace amine-associated receptors, breast cancer, dopamine, norepinephrine, epinephrine, histamine, serotonin, Gene Expression Omnibus, transcriptomic data, GPCR

## Abstract

Currently, the contribution of trace amine-associated receptors (TAARs) to breast cancer (BC) is recognized, but their associations with various pathological characteristics are not yet understood. There is accumulated transcriptomic data for BC tumors, which are represented in publicly accessible databases. We estimated TAARs’ (including TAAR1, TAAR2, TAAR5, TAAR6, TAAR8, and TAAR9) associations with BC stage, grade, and molecular subtypes in these data and identified that the expression of all TAARs was associated with more unfavorable cancer subtypes, including basal-like and HER2-positive tumors. Also, the significant upregulation of all TAARs was demonstrated in circulating tumor cells compared to the metastatic lesions. Considering that co-expressed genes are more likely to be involved in the same biologic processes, we analyzed genes that are co-expressed with TAARs in BC. These gene sets were enriched with the genes of the olfactory transduction pathway and neuroactive ligand–receptor interaction participants. TAARs are co-expressed with G-protein-coupled receptors of monoamine neurotransmitters including dopamine, norepinephrine, and serotonin as well as with other neuroactive ligand-specific receptors. Since TAAR1 is able to modulate the activity of monoamine receptors that are involved in the regulation of BC growth, TAAR1 and potentially other TAARs may be regarded as prospective therapeutic targets for breast cancer.

## 1. Introduction

The term “trace amines” refers to amines derived via the endogenous or bacterial decarboxylation of amino acids that are bioactive at concentrations below 50 ng/g (i.e., two orders lower than the amine neurotransmitters), which bind at these concentrations to one or more trace amine-associated receptors (TAARs). TAARs are a family of rhodopsin-like type A G-protein-coupled receptors (GPCR). Humans possess six TAARs types, including TAAR1, TAAR2, TAAR5, TAAR6, TAAR8, and TAAR9 [1,2]. TAAR1 and TAAR2 sense primary amines and TAAR5–9 are tuned toward diamines, polyamines, or tertiary amines [1]. These receptors were identified at first in the olfactory epithelium (except TAAR1), while TAAR1 was detected primarily in the brain. Later, all TAARs were also found in the brain structures, immune cells, gastrointestinal tract, kidney, testes, and other organs and tissues [1,3,4].

There is a growing appreciation of the potential contribution of TAARs in cancer development and progression [5,6,7,8,9]. Breast cancer (BC) accounted for approximately 24.5% of all cancer cases and 15.5% of cancer deaths in women, and the global burden of BC is rising in the world [10]. TAAR expression was identified in BC and higher levels of TAAR1, TAAR2, TAAR5, TAAR8, and TAAR9 mRNA in breast tumors are associated with better survival [5,7,9]. Treatment of the BC-derived MCF7 cell line with a high-affinity TAAR1 agonist 3-iodothyronamine (T1AM) [2] decreases cell viability and migration [6,11]. However, the role of TAAR1 in this effect remains unknown, since T1AM is internalized in tumor cells and co-localized with mitochondria [12]. Nevertheless, the cadaverine-elicited inhibition of cellular movement, the epithelial-to-mesenchymal transition, and metastases are suggested to be mediated by TAAR1, TAAR8, and TAAR9 [9].

TAAR1 is the most studied of the TAARs, and it is considered to regulate the other biogenic amine neurotransmitter receptors’ activation [1]. For example, beta2- adrenoreceptor (ADRA2A)/TAAR1 heterodimerization leads to the uncoupling of ADRA2A from the norepinephrine signaling pathway [13]. The modulation of the dopaminergic system by TAAR1 is mediated by dopamine D2 receptor (DRD2)/TAAR1 heterodimerization [14], which reduces β-arrestin 2 recruitment to DRD2 [2]. Additionally, TAAR1 activity alters the agonist potency at serotonin 5-HT1A (HTR1A) receptors [15].

Meanwhile, it is found that ligands of monoamine receptors, including ADRA2A, DRD2, and HTR1A, may demonstrate pro-apoptotic and antiproliferative effects in BC cells. Also, such compounds increase BC’s susceptibility to chemotherapeutic compounds and radiotherapy [16,17,18,19,20,21,22]. ADRA2A is downregulated in BC; its expression is associated with beneficial tumor characteristics [2,22] and the suppression of cell division [23,24,25]. Antipsychotics, which block D2 receptors, cause hyperprolactinemia, which promotes BC development [23,26,27]. In contrast, in BC patients, DRD2 antagonists may demonstrate the opposite effect, including the reduction in tumor growth and invasion and the induction of cell cycle arrest and apoptosis [17,19,22,28,29,30,31,32,33]. However, the DRD2 role in BC growth seems to be multifaceted. Bromocriptine, a DRD2 agonist, also could suppress proliferation and activate apoptosis in some BC-derived cell lines [20,34].

The complex modulation of monoamine signaling in tumors via targeting TAAR1 or other members of the TAAR family seems to be a prospective approach to managing BC. TAAR1 agonist Ulotaront is an investigational antipsychotic which demonstrated clinical efficacy in patients with schizophrenia without causing side effects like currently used medications [35,36]. Thus, the adaptation of TAAR ligands with minimal adverse effects for cancer treatment seems to be promising in perspective. The aim of this study is the evaluation of TAARs’ mRNA expression in BC and of associations between TAAR expression in tumors and its molecular characteristics.

## 2. Materials and Methods

### 2.1. Public Resources and Databases

The expression data were derived from the Gene Expression Omnibus (GEO) repository [37]. Three RNA-seq-generated datasets were selected for the study to estimate TAAR expression in primary breast tumors (GSE119937), metastatic samples (GSE113890, GSE184717), and circulating tumor cells (CTCs, GSE113890, refer to Table 1 for details). During the study, tumors in the GSE119937 dataset were classified under the PAM50 signature [38] using the genefu R package [39].

Also, we add a review of several microarray-generated datasets (refer to Table 2). Datasets that represent cohort studies which include between 301 and 683 participants were studied to estimate the association between tumor TNM stage (GSE131769, GSE102484, GSE20685, and GSE25066), grade (GSE58215, GSE25066, and GSE81002), and TAAR expression. Additionally, GSE88715 and GSE5847 were included in the study to evaluate the differences in TAAR expression between tumor cells and stroma.

### 2.2. Data Normalization and Statistical Analysis

Raw counts were normalized to count per million (CPM) using the edgeR package [40]. CPM values above the threshold level of 0.5 were considered positive [41]. The distribution of CPM-normalized expression levels in the analyzed samples was visualized using the beeswarm and the ggplot2 [42] R packages.

Each dataset was analyzed separately. The differently expressed genes were identified in accordance with the protocol “Molecular Profiling of RNA Tumors Using High-Throughput RNA Sequencing: From Raw Data to Systems Level Analyses” for the tumor profiling [43] via limma/voom analysis. Briefly, raw data were normalized using the voom function (voom is an acronym for variance modeling at the observational level). Differentially expressed genes were identified via the Bayes statistic test using the limma R package [44]. *p* values were adjusted for multiple testing corrections using the Benjamini–Hochberg method. Genes were considered differentially expressed if adjusted *p* values (P_adj_) < 0.05.

Each microarray-generated dataset was analyzed using the GEO2R interactive web-based interface available at http://www.ncbi.nlm.nih.gov/geo/geo2r/ (accessed on 20 December 2022). Differentially expressed genes were identified in the individual datasets between study subgroups, which were selected following the structure of the dataset’s Series Matrix File represented in the repository. The expression values were log2-normalized, if necessary, through the GEO2R auto-detect feature that checks the values of selected samples and automatically performs a log2 transformation on values determined not to be in log space [45].

### 2.3. Survival Analysis

The association of TAAR expression with disease outcome was estimated in the GSE20685 and GSE25066 microarray-generated datasets. Kaplan–Meier survival curves were used to show the association between TAAR expression and the time of death, recurrence, or metastatic recurrence. The plots were generated using the survminer R package. The statistical significance was estimated with the survival R package [46]. We used 5.0 as the cut-off value for log2-normalized gene expression to differentiate TAAR-positive and TAAR-negative tumor samples according to previous studies [47,48,49].

### 2.4. Gene Co-Expression Measurement

TAARs co-expressed genes were defined using Pearson’s correlation coefficient (r > 0.3, *p* < 0.05) for the CPM-normalized expression levels. Genes co-expressed with TAAR1, TAAR2, TAAR5, TAAR6, TAAR8, or TAAR9 in the different study groups were included in separate gene clusters. The comparative analysis of the selected clusters was performed as described below. The overlap between identified gene clusters was visualized with the VennDiagram R package [50].

### 2.5. KEGG Pathway Enrichment Analysis

KEGG pathway enrichment analysis (identification of the KEGG pathways that are significantly enriched by the genes of the selected set) was performed in the identified co-expressed gene clusters. The results were visualized using the clusterProfiler Bioconductor package [51]. We considered significant enrichment results only for KEGG pathways with a false discovery rate value of <0.05. Gene mapping on KEGG pathways was carried out using the KEGG Mapper web tool [52].

## 3. Results

### 3.1. TAARs’ mRNA Expression in Primary Breast Tumors, BC Metastases, and CTCs Is Confirmed via RNA-Seq-Generated Dataset Analysis

**Figure 1:**
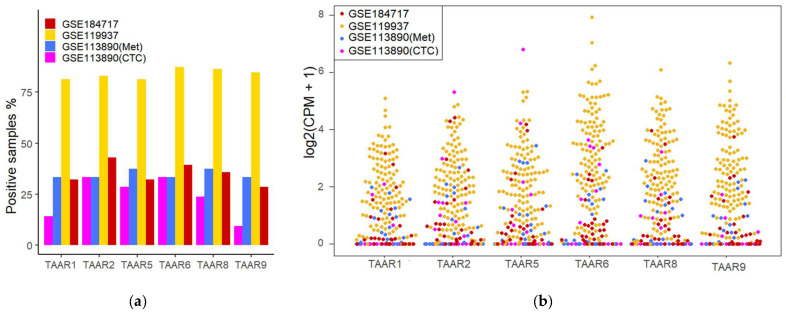
TAAR mRNA expression in primary breast tumors (GSE119937), metastatic breast tumors (GSE113890(Met), GSE184717), and circulating tumor cell (GSE113890(CTC)) samples: (**a**) the percent of samples expressing TAARs in RNA-seq datasets included in the analysis; (**b**) CPM-normalized expression levels of TAAR mRNA in breast cancer patients’ samples. CPM—count per million; CTC—circulating tumor cells; Met—metastases.

The analysis of transcriptomic RNA-generated datasets demonstrated that all known TAARs are expressed in BC tissues, both in primary BC samples and metastatic lesions. Applying the cut-off value CPM = 0.5, each TAAR mRNA was identified in at least 80% of the studied primary tumor samples (Figure 1a, Table S2). TAAR expression levels in primary tumor samples vary from 0 to 243.13 CPM (Figure 1b, Table S2).

Meanwhile, applying the cut-off value CPM = 0.5, each TAAR mRNA was identified at least in 28.6% of the studied metastatic tumor samples and in 9.5% of the circulating tumor cell samples (Figure 1a, Table S2). TAAR expression levels in metastatic tumor samples vary from 0 to 21.6 CPM (Figure 1b, Table S2) and from 0 to 111.51 CPM in CTC (Figure 1b, Table S2).

TAAR expression in primary tumors seems to be more pronounced than in metastatic lesions or circulating tumor cells. Considering that the data for different sample types were generated by distinct laboratories in dissimilar conditions, and GSE119937, representing primary tumor data, comprises samples with larger sequencing depth than in other datasets, we did not estimate this difference statistically to avoid batch bias.

### 3.2. TAAR Expression Pattern in BC Depends on Tumor Intrinsic Subtype but Is Not Associated with Tumor Grade or Stage

To identify the associations between TAAR expression and tumor characteristics like TNM stage or grade, we included several datasets that represent extensive cohort studies in our analysis. These studies include >300 patients per dataset, which allowed us to compare large subgroups and avoid bias. All selected studies were analyzed independently from each other to demonstrate the results’ reproducibility regardless of the cohort origin and the microarray platform applied for the study (Affymetrix Human Genome U133 Plus 2.0 Array or Illumina HumanHT-12 V3.0 expression beadchip data were compared when the association of tumor stage and TAAR expression was estimated). No associations with tumor T category (GSE20685, GSE102484), node status (GSE20685, GSE102484), metastatic status (GSE20685, GSE102484), stage (GSE131769), or differentiation grade (GSE58215, GSE80999) were identified. The lack of associations between TAAR expression and tumor T category, node status, or grade also was confirmed in the RNA-seq-generated dataset GSE119937.

**Figure 2:**
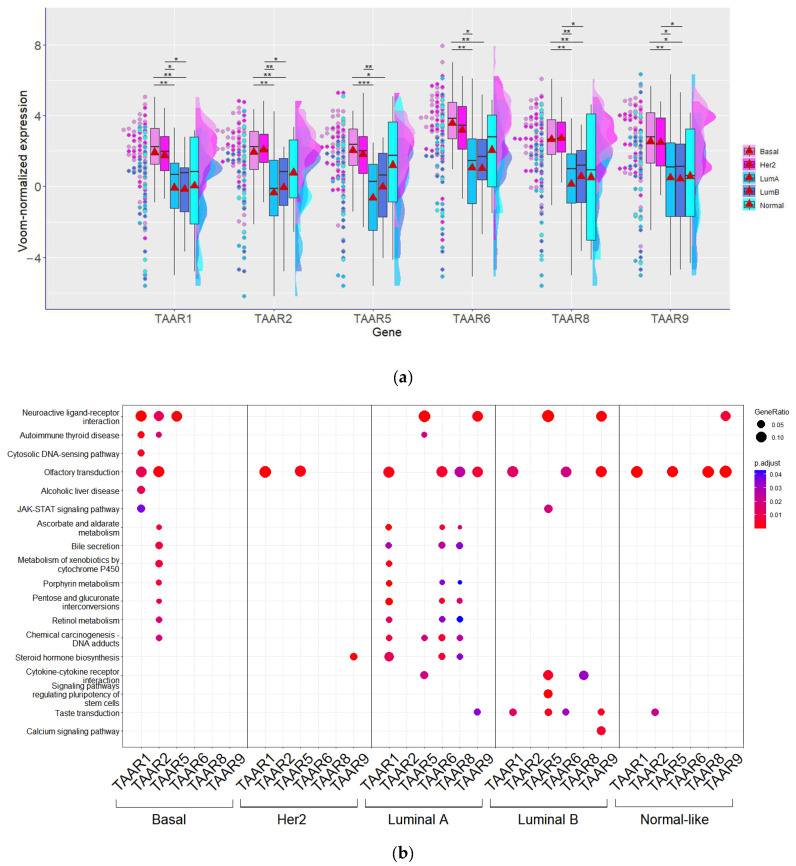
TAAR expression in different intrinsic subtypes of breast cancer (GSE119937). (**a**) Voom-normalized TAAR expression levels in basal-like (basal, *n* = 39), HER2-positive (Her2, *n* = 32), luminal A (LumA, *n* = 20), luminal B (LumB, *n* = 19), and normal-like (Norm, *n* = 8) breast tumors (GSE119937, tumors were classified via PAM50 signature and genefu R package); *—*p* < 0.05, **—*p* < 0.01, ***—*p* < 0.001; mean expression values are marked by triangle; (**b**) KEGG pathway enrichment analysis of top 1000 genes, co-expressed with TAARs in different intrinsic subtypes of breast cancer.

Meanwhile, when different intrinsic subtypes identified according to the PAM50 signature [45] in GSE119937 were compared, it was identified that all TAARs are significantly more expressed in basal-like tumors (*n* = 39) compared to luminal A (*n* = 20) or luminal B (*n* = 19) subtypes (Figure 2a). Likewise, all TAARs are more expressed in HER2-positive tumors (*n* = 32) compared to luminal A BC samples (Figure 2a). However, only TAAR1, TAAR2, TAAR8, and TAAR9 were expressed more in HER2-positive tumors compared to the luminal B subtype. For each TAAR gene, the top 1000 co-expressed genes were identified in each BC subtype, and enriched KEGG pathways in these gene clusters were compared (Figure 2b).

The results of the KEGG pathway enrichment analysis demonstrate the most complex associations between TAARs and biological processes in tumor tissues in luminal A BC, followed by luminal B and basal-like tumors. In normal-like BC, and especially in HER2-positive tumors, most functional associations of TAAR expression with other genes are lost. These results lead to the conclusion that the functional significance of TAAR expression seems to be impaired in these BC intrinsic subtypes. Meanwhile, in luminal A, luminal B, and basal-like tumors, subtype-specific associations were identified, which indicated possible differences in TAARs’ biological role in different molecular contexts. Nevertheless, in all BC subtypes, TAARs’ co-expressed genes are enriched with genes involved in olfactory transduction and neuroactive ligand–receptor interactions (Figure 2b).

### 3.3. TAAR Expression Levels and Functional Associations Differ Significantly between CTCs and Metastatic Lesions in BC Patients

**Figure 3:**
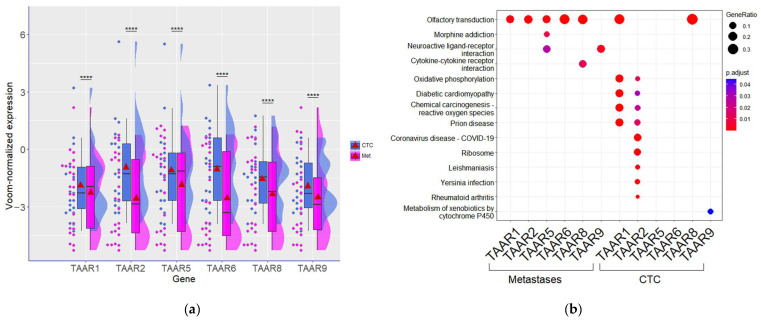
TAARs in metastatic breast tumors (*n* = 24) and circulating tumor cells (*n* = 21), isolated from the blood of breast cancer patients (GSE113890). (**a**) TAAR expression levels in metastatic breast tumors and circulating tumor cells; (**b**) KEGG pathway enrichment analysis of top 1000 genes co-expressed with TAARs in metastatic breast tumors and circulating tumor cells. ****—*p* < 0.0001.

The differences in TAAR expression levels were also estimated in CTCs isolated from metastatic BC patients and metastatic lesions samples from the same group. All TAARs were significantly higher expressed in CTCs compared to metastatic tumors (Figure 3a). This confirms the high heterogeneity of CTCs because the number of true-positive samples in this population is lower than in metastatic lesions (refer to Section 3.1).

Furthermore, the comparative analysis of KEGG pathway enrichment in the clusters of top 1000 genes co-expressed with each TAAR in each sample type demonstrates that in metastatic tumors, TAARs are predominantly co-expressed with olfactory transduction pathway components. Also, a less pronounced association with neurotransmitter systems components (including the “Neuroactive ligand–receptor interaction” pathway and “Morphine addiction” pathway) was identified (Figure 3b). In contrast, in CTCs, other pathways are co-activated with TAAR expression. The identified differences may mirror significant differences between CTCs and tumors, accompanied by higher TAAR expression and changes in the repertoire of signal pathways that these receptors can influence.

### 3.4. TAAR Expression Is Associated with Cancer Epithelial Cells Rather Than Stromal Cells

**Figure 4:**
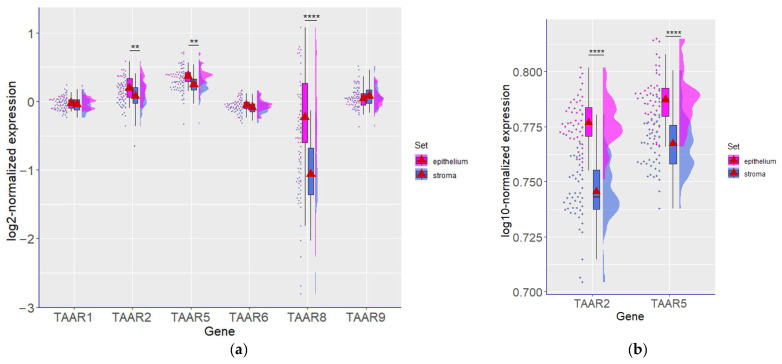
TAAR mRNA expression in tumor epithelium and stroma. (**a**) GSE88715 dataset’s TAAR expression pattern, (**b**) GSE5847 dataset’s TAAR expression pattern. **—*p* < 0.01, ****—*p* < 0.0001.

To confirm that TAARs expression in tumors samples reflects TAAR mRNA levels in tumor cells and is not dependent on TAAR mRNA from other cell types in tumor tissue, we analyzed the expression levels of TAAR in datasets GSE88715 and GSE5847. Both these datasets comprise expression profiles identified in micro-dissected tumor samples, which represent tumor cells or stroma separately. Because of the lack of RNA-seq-generated data with relevant sequencing depth, we apply the above-mentioned microarray-generated datasets (GSE5847 represents data only for TAAR2 and TAAR5; Figure 4a,b).

The expression of TAAR2, TAAR5, and TAAR8 was significantly higher in neoplastic epithelial cells compared to stromal structures (Figure 4a,b). Other TAARs, i.e., TAAR1, TAAR6, and TAAR9, are expressed in the neoplastic epithelium and tumor stroma at the same levels.

Since the GSE5847 dataset includes data for non-inflammatory and inflammatory tumors, we also compare the TAAR expression between these two BC forms. No differences in TAAR expression were identified between inflammatory and non-inflammatory tumors, both in epithelial and stromal compartments. Meanwhile, the statistically significant difference in TAAR expression between the epithelium and stroma is reproducible in both forms of BC.

### 3.5. TAARs mRNA Expression Is Not Associated with Outcome in Studied Microarray-Generated Datasets

To study the associations between TAARs expression and the tumor response to treatment, we estimate the patients’ follow-up in two datasets. In the study represented in the GSE20685 dataset, the patients were treated with adjuvant chemotherapy (predominately a cyclophosphamide, methotrexate, and fluorouracil combination or a cyclophosphamide, doxorubicin, fluorouracil combination) or hormone therapy. The dataset design permits us to estimate TAAR expression’s association with overall survival, recurrence-free survival, and metastases-free survival. No significant associations were identified between TAAR expression and survival or recurrence for patients who received chemotherapeutic medication or hormonal therapy (refer to Supplementary S2 for Kaplan–Meier analysis results).

We analyze relapse-free survival in patients who received neoadjuvant taxane–anthracycline chemotherapy, represented in the dataset GSE25066. No associations between disease progression after the treatment and TAAR2 or TAAR5 expression were identified (no data for other TAARs were represented in the dataset because of the limitations of the applied microarray platform).

### 3.6. KEGG Pathway Enrichment Analysis Demonstrates TAARs’ Co-Expression with Neuroactive Ligand-Binding GPCRs, Including Monoamine Receptors

To precisely determine genes co-expressed with TAARs for further analysis, we compare gene sets which are co-expressed with each TAAR gene in three studied RNA-seq-generated datasets, i.e., GSE119937, GSE113890 (only metastatic lesion expression was considered), and GSE184717. This approach permits us to select the genes which are co-expressed with TAARs both in primary tumors in different development stages and metastatic lesions.

**Figure 5:**
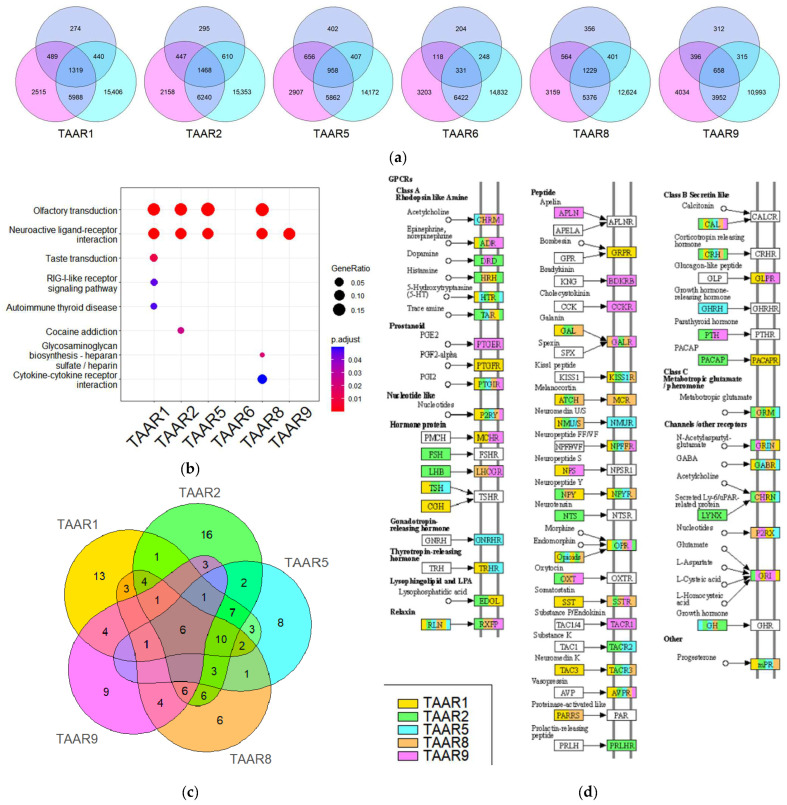
Functional analysis of genes co-expressed with TAARs in primary and metastatic breast tumors. (**a**) Venn diagram illustrating an overlay of genes co-expressed (r > 0.3, *p* > 0.05) with TAARs in primary (GSE119937, blue circle) and metastatic breast tumors (GSE113890, GSE184717, magenta and cyan circles, respectively); (**b**) KEGG pathway enrichment analysis of genes, co-expressed with TAARs in all three datasets; (**c**) Venn diagram illustrating an overlay of genes co-expressed with TAARs in all three datasets and involved in the “Neuroactive ligand–receptor interactions” KEGG pathway and (**d**) mapping these genes on the “Neuroactive ligand–receptor interactions” KEGG pathway scheme.

The genes co-expressed with TAARs at a significant level (r > 0.3, *p* < 0.05) were selected in each dataset, and the overlap between these gene clusters was estimated. It should be noted that in GSE119937, the clusters of genes co-expressed with each TAAR gene are narrower than in other datasets. It may be related to the higher sequencing depth in this dataset, and, as a result, more specific data for the low-expressed genes’ mRNA quantity. Despite this discrepancy, most genes co-expressed with any of the TAARs in GSE119937 also co-expressed with the same TAAR in other datasets (Figure 5a).

The genes, which are co-expressed with the same TAAR gene in all three datasets, were grouped in the corresponding clusters (i.e., TAAR1 cluster for the genes co-expressed with TAAR1 in three datasets, TAAR2 cluster for the genes co-expressed with TAAR2 in three datasets, etc.).

The KEGG pathway enrichment analysis provides positive results for all TAAR receptors’ genes except TAAR6. TAAR1, TAAR2, TAAR5, TAAR8, and TAAR9 RNA expression levels in all three datasets were correlated with the mRNA content of genes involved in the “Neuroactive ligand–receptor interactions” KEGG pathway. Also, TAAR1, TAAR2, TAAR5, and TAAR8 co-expression with “Olfactory transduction” KEGG pathway components was identified (Figure 5b).

As the receptors of neuroactive ligands are suggested to be perspective targets for cancer treatment, we examined the association between TAARs and neuroactive ligand–receptor signaling in more detail. Considerable differences were revealed in the repertoires of ”Neuroactive ligand–receptor interactions” KEGG pathway components in the compared TAAR1, TAAR2, TAAR5, TAAR8, and TAAR9 clusters. Only six genes of the ”Neuroactive ligand–receptor interactions” KEGG pathway, including opioid receptor kappa 1 (OPRK1), neuropeptide FF receptor 1 (NPFFR1), calcitonin-related polypeptide alpha (CALCA), prostaglandin I2 receptor (PTGIR), arginine vasopressin receptor 2 (AVPR2), and orphan receptor GPR35, are co-expressed with all five TAARs (Figure 5c).

The mapping of ”Neuroactive ligand–receptor interactions” KEGG pathway components identified in the TAAR1, TAAR2, TAAR5, TAAR8, and TAAR9 clusters demonstrated these receptors are co-expressed with genes coding multiple receptors including but not limited to rhodopsin-like GPCRs and neuroactive proteins, which involved in the number signaling pathways, including monoaminergic neurotransmission, peptide, hormone, prostaglandin, and other signaling molecule pathways (Figure 5d).

## 4. Discussion

In line with previously published evidence, the expression of all TAARs was identified in BC tumor tissue samples [5,7,9]. No associations were demonstrated between tumor size or metastatic potential and TAAR expression in several large cohort studies which were analyzed in parallel. However, significantly higher TAAR expression was identified in tumors with less favorable molecular subtypes, i.e., basal-like and HER2-positive BC. Common BC categorization is based on the presence or absence of estrogen or progesterone receptors and human epidermal growth factor 2 (ERBB2; formerly HER2). On this basis, breast tumors are classified into luminal A, luminal B, HER2-positive, and triple-negative groups with differences in prognosis and treatment regimen [53,54]. These intrinsic subtypes are associated with specific expression profiles, genetic abnormalities, and epigenetic features [55]. Patients with non-metastatic hormone receptor-positive luminal A or luminal B tumors receive endocrine therapy, and a minority of HER2-expressing luminal B cancers receive chemotherapy as well. In patients with HER2-positive tumors, HER2-targeted antibody or small-molecule inhibitor therapy combined with chemotherapy is administrated. Patients with triple-negative tumors receive chemotherapy alone. This phenotype is associated with the worst recurrence and survival prognosis [38,54,55]. Normal-like breast carcinomas do not seem to make up a true subtype, since some tumors may be characterized as normal-like because of contamination with normal tissue [56].

The differential distribution of monoamine receptors in different BC intrinsic subtypes was described in an example of DRD2, which is more common in HER2-negative patients than in HER2-positive patients [57]. The data about TAAR1 expression in different BC intrinsic subtypes are controversial. Pitts and coauthors [58] demonstrate that the level of TAAR1 expression does not appear to correlate with known molecular phenotypes of BC. Yet, TAAR1 expression’s association with HER2 positivity was described by Vattai and colleagues [7]. Our analysis identified that different intrinsic subtypes of BC demonstrate profound differences in TAAR mRNA expression levels and the patterns of genes co-expressed with TAARs [58]. The most intractable basal-like breast tumors are characterized by the overexpression of proliferation- and cell-stemness-related genes [53,54,55]. Moreover, this subtype is characterized by high heterogeneity [58]. The stable expression of TAAR1 and other TAARs in this group allows us to consider them as possible therapeutic targets in basal-like BC. In basal and HER2-positive tumors, which express TAARs at higher levels, the enrichment of these genes in KEGG pathways is less complex than in luminal A/B cancers, which express TAARs at lower levels.

The present study identified TAAR expression in metastatic breast tumors. The determined expression levels were lower than in primary tumors, but we did not find it possible to compare these groups because the data were obtained by different study groups with distinct equipment and protocols. Instead, we form a comparative analysis of TAAR-co-expressed genes in metastatic lesions and CTCs. We identified the similarity of KEGG pathways enriched in TAAR-co-expressed gene clusters in metastatic lesions and primary tumors, at least at the key points. In both groups, TAARs were co-expressed with genes involved in olfactory transduction and neuroactive ligand–receptor interactions. The loss of complex association patterns identified in luminal A/B primary tumors may be the consequence of changes in intrinsic tumor subtype, which is frequently observed in metastases compared to primary tumors, especially in luminal A/B cases [59,60].

Meanwhile, in CTCs, TAAR expression levels were significantly higher than in metastatic lesions. At the same time, the association of TAAR2, TAAR5, TAAR6, and TAAR8 with olfactory transduction is lost, and several new associations appear. Thus, in CTCs, TAAR1 and TAAR2 become co-expressed with genes involved in oxidative phosphorylation and reactive oxygen species (ROS) production. We identified three KEGG pathways enriched in the set of genes that are co-expressed with TAAR1 in CTC, for which the enrichment core (the leading subset of genes that contribute most to the enrichment score) includes cytochromes, i.e., “Oxidative phosphorylation”, “Diabetic cardiomyopathy”, and “Prion diseases”. For the KEGG pathways “Diabetic cardiomyopathy” and “Prion diseases”, enrichment cores also include NADPH oxidase. Also, TAAR1 and TAAR2 are co-expressed with genes involved in carcinogenesis, and TAAR2 is co-expressed with genes that are coding components of the immune response. CTCs form the heterogeneous low-density population of tumor cells in circulation [61]. Higher CTCs levels in circulation are associated with worse outcomes [62]. Global differences in BC-related gene expression and a decrease of ER, PR, and HER2 expression levels in CTCs compared with primary tumors were identified [63]. These distinctions may reflect the vulnerability of CTCs to various damaging factors, including anoikis, oxidative stress, and immune attack [64]. Also, before growing into metastases, tumor cells that escape destruction may remain in a dormant state for a long time [65]. The significant changes in TAAR expression and expected functional associations may be related to the unique characteristics of this malignant cell population.

The identified differences in TAAR2 and TAAR5 expression levels between tumor cells and stroma seem to be reproducible in different groups, including inflammatory cancer, whilst tumorous and stromal cells demonstrate similar expression levels of TAAR1, TAAR6, and TAAR8. The stromal tumor compartment comprises resident cancer-associated fibroblasts (CAFs), endothelial cells, pericytes, adipose tissue, macrophages, neutrophils, mast cells, and T- and B-lymphocytes [66,67]. Notably, CAFs are a heterogeneous population, including myofibroblastic CAFs, CAFs with elevated expression of inflammatory cytokines or the MHCII complex, and other groups [68]. CAFs are derived from normal fibroblasts [69], but part of the population may arise from cancer cells via epithelial–mesenchymal transition [70,71]. Currently, the expression pattern of TAARs in CAFs remains undiscovered, and immune-cell-infiltrating tumors seem to be the most probable source of TAAR mRNA, including TAAR2 [72,73] and TAAR5 [74] in tumor stroma. Also, it cannot be ruled out that the identified difference in the expression of TAAR2 and TAAR5 mirrors the loss of cancer-promoting pathways in tumor stroma. However, the received results allow us to suppose that TAAR1 ligands or the ligands of other TAARs may regulate biological processes not only in BC cells but also in malignant tumor stroma.

In the present study, we could not identify an association between TAAR expression and survival, which was described previously by other authors [5,7,9], possibly because of the low sensitivity of microarrays, which were applied to generate the analyzed datasets. More precise RNA-seq-generated data with relevant sequencing depth are not available currently; therefore, the study of TAAR expression’s impact on the effectiveness of different treatment schemes remains the problem of further research.

The analysis of the genes which are co-expressed with different TAARs identified an association with the “Olfactory transduction” pathway, especially with ORs, like it was described previously in melanoma [75]. Despite odorant receptors (ORs) primarily being identified in the olfactory epithelium, currently, these receptors were observed outside of the olfactory system in all other human tissues tested to date [73,74]. This is the largest gene family in the mammalian genome, and in humans, more than 400 OR genes were identified [76]. ORs are commonly overexpressed in tumor tissues, including BC, compared to adjacent noncancerous samples [75,76,77,78,79,80,81]. Different OR expression may demonstrate various associations with tumor characteristics. For example, OR2W3 upregulation is associated with decreased survival probability [82], and OR51J1 is overexpressed in HER2-enriched and triple-negative tumors compared to luminal BC but does not correlate with tumor morphologic characteristics [77]. Functional evaluations have shown that ORs may regulate cancer cell invasiveness, metastasis, (de)differentiation, proliferation, and apoptosis in BC [79].

Also, in BC, all TAARs except TAAR6 were co-expressed with a variety of other neuroactive ligand-recognizing molecules. TAAR1 interactions with epinephrine [13] and 5-HT [15] signaling, which are involved in BC development and progression [18,23,25,75,83,84,85,86,87] are previously described. The co-expression of these receptors in tumors supports the possibility of modulating epinephrine and 5-HT signals in tumor tissue by TAAR1. Additionally, TAAR1 interacts with DRD2 and acts as the rheostat of DRD2-mediated dopamine signaling. DRD2 is known to be expressed in BC, and its antagonists demonstrate an antiproliferative effect in BC cells [28,34]; thus, the modulation of DRD2 activity by TAAR1 ligands also may be considered as a perspective approach to the treatment of this disease.

Also, the co-expression of TAAR1 and other TAARs with histamine receptors was identified. All histamine receptors are expressed in BC and are suggested to be involved in tumor growth regulation [88,89]. Other rhodopsin-like receptors were also co-expressed with several TAARs, including muscarinic receptors, whose stimulation leads to BC cell growth [84,90]. These receptors are expressed in malignant breast tumors but not in normal breast tissue [84].

The components of numerous non-amine neuroactive ligand signaling pathways, for which co-expression with TAARs in BC samples was confirmed in the present study, were previously identified as probable prognostic markers or therapeutic targets for mammary tumors. For example, prostanoid receptors PTGER2 and PTGER4 [77] are negatively associated with the disease outcome. Neuropeptides like neuropeptide Y and substance P are involved in BC progression [85,87]. The galaninergic system has been observed in BC, but its impact on BC progression remains unidentified. It is notorious that the effect of galanin on tumor growth may be both stimulatory (like in colorectal tumors) and inhibitory (like in gastric cancer) [91]. Meanwhile, somatostatin receptors, which also are co-expressed with various TAARs, are more often presented in tumor tissue in patients with better outcomes [92] and may be considered a therapeutic target in BC [93]. The interaction between TAARs and these GPCR-mediated signaling systems is not described; however, it shall be not excluded.

This study has several limitations. (i) The first and main limitation is that the transcriptomic datasets are generated with different sequencing depths and distinct protocols, so the possibilities to compare different types of cancer tissues described in this article were restricted. (ii) Only a few datasets in the GEO repository were relevant for the study, so we were required to analyze the association between TAAR expression and tumor pathological characteristics like grade or TNM stage in microarray data instead of more precise RNA-seq data. (iii) The current study identified some associations between TAAR expression and the expression of some other receptors involved in BC pathogenesis, but these conclusions are based on statistical simulations and do not provide information on the actual interactions between these receptors. (iv) mRNA abundance is only an indirect indicator of downstream expression because the resulting protein expression and its functional activity level depend on multiple factors, including RNA stability, modifications, the translation rate and protein turnover, localization in the cell, and the availability of ligands or co-interacting proteins. Also, the impact of germline genetic features and somatic aberrations in tumors affecting gene expression were not taken into account in the present study [94]. (v) In addition, our conclusions lack experimental validation, which was out of the scope of this study, and the work provides only preliminary evidence of TAARs’ role in BC.

Breast tumor development is associated with the deregulation of the local monoamine metabolism [23,95] and their receptors’ expression [23,96]. We found that TAARs are expressed in breast tumors and apparently are co-regulated with other monoamine receptors. Since the anticancer effect of monoamine receptors’ ligands was previously identified in BC, TAAR1 seems to be a prospective therapeutic target in BC as the modulator of monoamine signaling. This receptor is expressed in basal-like tumors, for which therapeutic options are limited.

## 5. Conclusions

The analysis of public transcriptomic data indicated the possible involvement of trace amine-associated receptors in breast cancer growth. All TAAR genes identified in humans are expressed in primary breast tumors, metastatic tumors, and CTCs. Differential gene expression analysis revealed a non-homogeneous distribution of TAAR expression in different molecular subtypes of breast cancer, so the molecular context has more influence on TAAR expression than tumor progression. Also, the involvement of TAARs in different signaling pathways varies significantly between different BC subtypes. The identified loss of functional associations between TAARs and other genes in basal-like and HER2-positive tumors compared with luminal A BC samples, identified through correlation and KEGG pathway enrichment analysis, is not quite typical because the up-regulation of expression accompanies it. The overlap between genes co-expressed with TAARs in primary tumors and metastatic lesions is significant, and we found correlations between the expression levels of TAAR1, TAAR2, TAAR5, TAAR8, and TAAR9 and genes associated with neuroactive ligand signaling in both kinds of neoplasms. These preliminary data suggest that TAAR1 and other TAARs are expressed in BC and, thus, targeting these receptors may modulate the function of other monoamine receptors and possibly other GPCR receptor–ligand systems in malignant breast tissue. Further experimental studies are now needed for the confirmation of these associations and the elucidation of the underlying mechanisms.

## Figures and Tables

**Table 1:** Characteristics of RNA-seq-generated datasets included in the review.

Dataset ID	Title	*n*	Samples’ Characteristics
GSE113890	The whole transcriptional landscape of circulating tumor cells compared to metastases in stage IV breast cancer	45	Stage IV patients, 21 circulating tumor cells samples and 24 metastatic lesions were analyzed
GSE119937	Molecular Determinants of Post-Mastectomy Breast Cancer Recurrence	118 ^1^	Primary breast cancer samples, grade I–III (refer to Table S1 for details)
GSE184717	Novel temporal and spatial patterns of metastatic colonization from breast cancer rapid-autopsy tumor biopsies	28	Breast cancer metastatic lesions

^1^ The number of samples which correspond to the aims of the study and included in the analysis; to prevent overload with false-negative results, only samples with the reads number in SRA files over 37.5 million were included in the analysis of the GSE113799 dataset.

**Table 2:** Characteristics of microarray-generated datasets included in the review.

Dataset ID	Title	*n*	Samples Characteristics	Platform
GSE5847	Tumor and stroma from breast by LCM	95	47 stromal microdissected samples, 48 epithelial microdissected samples from 15 invasive and 35 non-invasive breast cancers	Affymetrix Human Genome U133A Array
GSE20685	Microarray-based molecular subtyping of breast cancer	327	Primary breast cancer samples, Stage I–III (refer to Table S1 for details)	Affymetrix Human Genome U133 Plus 2.0 Array
GSE25066	Genomic predictor of response and survival following neoadjuvant taxane-anthracycline chemotherapy in breast cancer	508	Primary breast cancer samples, Stage I–III (refer to Table S1 for details)	Affymetrix Human Genome U133A Array
GSE58215	Integrated analysis reveals microRNA networks coordinately expressed with key proteins in breast cancer	566	Primary breast cancer samples, grade I–III (refer to Table S1 for details)	Agilent-028004 SurePrint G3 Human GE 8x60K Microarray
GSE80999	Integrative clustering reveals a novel split in the luminal A subtype of breast cancer with impact on outcome	356	Primary breast cancer samples, grade I–III (refer to Table S1 for details)	Agilent-028004 SurePrint G3 Human GE 8x60K Microarray
GSE88715	Gene expression profiles of microdissected tumor epithelium and stroma from TN breast tumors	76	38 triple negative breast cancer samples, tissue compartments isolated by laser capture microdissection	Agilent-028004 SurePrint G3 Human GE 8x60K Microarray
GSE102484	Expression data from invasive breast cancer patient	683	Primary breast cancer samples, Stage I–III (refer to Table S1 for details)	Affymetrix Human Genome U133 Plus 2.0 Array
GSE131769	Genomic signature of the standardized uptake value in 18F-fluorodeoxyglucose positron emission tomography in breast cancer	301	Surgically resected breast cancer, Stage I–III (refer to Table S1 for details)	Illumina HumanHT-12 V3.0 expression beadchip

## Data Availability

All datasets are available in the GEO database (https://www.ncbi.nlm.nih.gov/geo/, accessed on 4 September 2023).

## Supplementary Materials

The following supporting information can be downloaded at: https://www.mdpi.com/article/10.3390/biom13091361/s1, Supplementary S1: Tables S1 and S2; Supplementary S2: association of TAARs expression in breast carcinoma with disease outcome; Supplementary S3: association of TAARs expression in breast carcinoma disease recurrence after the treatment including neoadjuvant taxane-anthracycline chemotherapy.
