# Supplementary material for: The Expression of Trace Amine-Associated Receptors (TAARs) in Breast Cancer Is Coincident with the Expression of Neuroactive Ligand–Receptor Systems and Depends on Tumor Intrinsic Subtype
Source: Biomolecules. 2023 Sep 7;13(9):1361. doi: 10.3390/biom13091361 (PMC10526748; doi:10.3390/biom13091361)
Supplement: Supplementary file 1 [file biomolecules-13-01361-s001.zip › Supplementary S1.pdf]

Supplementary S1: Tables S1 and S2

Table S1 Clinicopathological characteristics of breast cancer samples in datasets included in the analysis of associations between clinico-pathologic breast cancer charactristics and TAARs expression*

Dataset ID	GSE20685	GSE25066	GSE58215	GSE80999	GSE102484	GSE119937	GSE131769
Tumor's size and extent							
T1	99	30	ND	ND	276	61	ND
T2	189	255	ND	ND	377	51	ND
T3	26	145	ND	ND	24	4	ND
T4	12	75	ND	ND	0	0	ND
Lymph node involvement							
N0	137	157	ND	ND	300	58	ND
N1	87	244	ND	ND	214	60	ND
N2	63	66	ND	ND	87	0	ND
N3	39	41	ND	ND	82		ND
Distant metastases							
M0	319	508	ND	ND	582	ND	ND
M1	8	0	ND	ND	101	ND	ND
Disease stage							
Stage I	ND	8	ND	ND	175	ND	77
Stage II	ND	272	ND	ND	328	ND	157
Stage III	ND	224	ND	ND	174.	ND	62
Stage IV	ND	0	ND	ND	0	ND	0
Tumor differentiation (grade)							
G1	ND	32	43	52	ND	3	ND
G2	ND	190	109	151	ND	23	ND
G3	ND	284	283	153	ND	80	ND

* The data are represented in accordance with series data from the GEO database and may be lost for the part of samples in the study groups.

Table S2 The levels and frequency of TAARs mRNA expression in breast cancer samples described in RNA-seq generated datasets included in the analysis (in CPM).

Dataset ID		GSE119937	GSE184717	GSE113890	GSE113890
Sample characteristic		Primary tumor	Metastases	Metastases	CTC
TAAR1	min	0	0	0	0
	max	33.37	7.97	2.97	3.28
	mean	4.32	0.88	0.60	0.29
	% of positive	81.4	32.1	33.3	14.3
TAAR2	min	0	0	0	0
	max	28.45	20.60	5.43	38.77
	mean	4.93	2.28	0.82	2.54
	% of positive	83.1	42.9	33.3	33.3
TAAR5	min	0	0	0	0
	max	39.18	17.27	9.90	111.51
	mean	5.23	1.68	1.48	6.53
	% of positive	81.4	32.8	37.5	28.6
TAAR6	min	0	0	0	0
	max	242.12	9.30	4.94	11.54
	mean	15.40	1.06	0.89	1.99
	% of positive	87.3	39.3	33.3	33.3
TAAR8	min	0	0	0	0
	max	67.02	14.61	6.59	8.34
	mean	7.74	1.41	0.90	0.68
	% of positive	86.4	35.7	37.5	23.8
TAAR9	min	0	0	0	0
	max	79.98	12.51	3.76	2.31
	mean	8.20	1.12	0.68	0.18
	% of positive	84.7	28.6	33.3	9.5
