# Supplementary material for: The Expression of Trace Amine-Associated Receptors (TAARs) in Breast Cancer Is Coincident with the Expression of Neuroactive Ligand–Receptor Systems and Depends on Tumor Intrinsic Subtype
Source: Biomolecules. 2023 Sep 7;13(9):1361. doi: 10.3390/biom13091361 (PMC10526748; doi:10.3390/biom13091361)
Supplement: Supplementary file 1 [file biomolecules-13-01361-s001.zip › Supplementary S2.html]

Supplementary 2: association of TAARs expression in breast carcinoma with disease outcome


# Supplementary 2: association of TAARs expression in breast carcinoma with disease outcome

Here, the analysis of survival for patients described in the GSE20685
dataset is summarized. We applied the threshold log2 expression = 5.0 to
classify tumor samples as TAAR-positive or TAAR-negative for each TAAR
gene expression. Time is represented in years.

##### **Association of TAARs expression and overall survival**

```
## 
##  Pairwise comparisons using Log-Rank test 
## 
## data:  KM and TAAR1exp + treatment 
## 
##                                       TAAR1exp=Negative, treatment=Chemo   
## TAAR1exp=Negative, treatment=Hormonal 0.31                                 
## TAAR1exp=Positive, treatment=Chemo    0.42                                 
## TAAR1exp=Positive, treatment=Hormonal 0.31                                 
##                                       TAAR1exp=Negative, treatment=Hormonal
## TAAR1exp=Negative, treatment=Hormonal -                                    
## TAAR1exp=Positive, treatment=Chemo    0.27                                 
## TAAR1exp=Positive, treatment=Hormonal 0.77                                 
##                                       TAAR1exp=Positive, treatment=Chemo   
## TAAR1exp=Negative, treatment=Hormonal -                                    
## TAAR1exp=Positive, treatment=Chemo    -                                    
## TAAR1exp=Positive, treatment=Hormonal 0.27                                 
## 
## P value adjustment method: BH
```

```
## 
##  Pairwise comparisons using Log-Rank test 
## 
## data:  KM and TAAR2exp + treatment 
## 
##                                       TAAR2exp=Negative, treatment=Chemo   
## TAAR2exp=Negative, treatment=Hormonal 0.38                                 
## TAAR2exp=Positive, treatment=Chemo    0.50                                 
## TAAR2exp=Positive, treatment=Hormonal 0.38                                 
##                                       TAAR2exp=Negative, treatment=Hormonal
## TAAR2exp=Negative, treatment=Hormonal -                                    
## TAAR2exp=Positive, treatment=Chemo    0.38                                 
## TAAR2exp=Positive, treatment=Hormonal 0.92                                 
##                                       TAAR2exp=Positive, treatment=Chemo   
## TAAR2exp=Negative, treatment=Hormonal -                                    
## TAAR2exp=Positive, treatment=Chemo    -                                    
## TAAR2exp=Positive, treatment=Hormonal 0.38                                 
## 
## P value adjustment method: BH
```

```
## 
##  Pairwise comparisons using Log-Rank test 
## 
## data:  KM and TAAR5exp + treatment 
## 
##                                       TAAR5exp=Negative, treatment=Chemo   
## TAAR5exp=Negative, treatment=Hormonal 0.25                                 
## TAAR5exp=Positive, treatment=Chemo    0.98                                 
## TAAR5exp=Positive, treatment=Hormonal 0.82                                 
##                                       TAAR5exp=Negative, treatment=Hormonal
## TAAR5exp=Negative, treatment=Hormonal -                                    
## TAAR5exp=Positive, treatment=Chemo    0.25                                 
## TAAR5exp=Positive, treatment=Hormonal 0.98                                 
##                                       TAAR5exp=Positive, treatment=Chemo   
## TAAR5exp=Negative, treatment=Hormonal -                                    
## TAAR5exp=Positive, treatment=Chemo    -                                    
## TAAR5exp=Positive, treatment=Hormonal 0.82                                 
## 
## P value adjustment method: BH
```

```
## 
##  Pairwise comparisons using Log-Rank test 
## 
## data:  KM and TAAR8exp + treatment 
## 
##                                       TAAR8exp=Negative, treatment=Chemo   
## TAAR8exp=Negative, treatment=Hormonal 0.26                                 
## TAAR8exp=Positive, treatment=Chemo    0.78                                 
## TAAR8exp=Positive, treatment=Hormonal 0.26                                 
##                                       TAAR8exp=Negative, treatment=Hormonal
## TAAR8exp=Negative, treatment=Hormonal -                                    
## TAAR8exp=Positive, treatment=Chemo    0.26                                 
## TAAR8exp=Positive, treatment=Hormonal 0.59                                 
##                                       TAAR8exp=Positive, treatment=Chemo   
## TAAR8exp=Negative, treatment=Hormonal -                                    
## TAAR8exp=Positive, treatment=Chemo    -                                    
## TAAR8exp=Positive, treatment=Hormonal 0.26                                 
## 
## P value adjustment method: BH
```

```
## 
##  Pairwise comparisons using Log-Rank test 
## 
## data:  KM and TAAR9exp + treatment 
## 
##                                       TAAR9exp=Negative, treatment=Chemo   
## TAAR9exp=Negative, treatment=Hormonal 0.65                                 
## TAAR9exp=Positive, treatment=Chemo    0.82                                 
## TAAR9exp=Positive, treatment=Hormonal 0.42                                 
##                                       TAAR9exp=Negative, treatment=Hormonal
## TAAR9exp=Negative, treatment=Hormonal -                                    
## TAAR9exp=Positive, treatment=Chemo    0.48                                 
## TAAR9exp=Positive, treatment=Hormonal 0.67                                 
##                                       TAAR9exp=Positive, treatment=Chemo   
## TAAR9exp=Negative, treatment=Hormonal -                                    
## TAAR9exp=Positive, treatment=Chemo    -                                    
## TAAR9exp=Positive, treatment=Hormonal 0.42                                 
## 
## P value adjustment method: BH
```

##### **Association of TAARs expression and recurrence-free survival**

```
## 
##  Pairwise comparisons using Log-Rank test 
## 
## data:  KM and TAAR1exp + treatment 
## 
##                                       TAAR1exp=Negative, treatment=Chemo   
## TAAR1exp=Negative, treatment=Hormonal 0.12                                 
## TAAR1exp=Positive, treatment=Chemo    0.93                                 
## TAAR1exp=Positive, treatment=Hormonal 0.76                                 
##                                       TAAR1exp=Negative, treatment=Hormonal
## TAAR1exp=Negative, treatment=Hormonal -                                    
## TAAR1exp=Positive, treatment=Chemo    0.12                                 
## TAAR1exp=Positive, treatment=Hormonal 0.63                                 
##                                       TAAR1exp=Positive, treatment=Chemo   
## TAAR1exp=Negative, treatment=Hormonal -                                    
## TAAR1exp=Positive, treatment=Chemo    -                                    
## TAAR1exp=Positive, treatment=Hormonal 0.76                                 
## 
## P value adjustment method: BH
```

```
## 
##  Pairwise comparisons using Log-Rank test 
## 
## data:  KM and TAAR2exp + treatment 
## 
##                                       TAAR2exp=Negative, treatment=Chemo   
## TAAR2exp=Negative, treatment=Hormonal 0.34                                 
## TAAR2exp=Positive, treatment=Chemo    0.41                                 
## TAAR2exp=Positive, treatment=Hormonal 0.38                                 
##                                       TAAR2exp=Negative, treatment=Hormonal
## TAAR2exp=Negative, treatment=Hormonal -                                    
## TAAR2exp=Positive, treatment=Chemo    0.37                                 
## TAAR2exp=Positive, treatment=Hormonal 0.38                                 
##                                       TAAR2exp=Positive, treatment=Chemo   
## TAAR2exp=Negative, treatment=Hormonal -                                    
## TAAR2exp=Positive, treatment=Chemo    -                                    
## TAAR2exp=Positive, treatment=Hormonal 0.38                                 
## 
## P value adjustment method: BH
```

```
## Warning in pchisq(chi, df, lower.tail = FALSE): NaNs produced
```

```
## 
##  Pairwise comparisons using Log-Rank test 
## 
## data:  KM and TAAR5exp + treatment 
## 
##                                       TAAR5exp=Negative, treatment=Chemo   
## TAAR5exp=Negative, treatment=Hormonal 0.20                                 
## TAAR5exp=Positive, treatment=Chemo    0.20                                 
## TAAR5exp=Positive, treatment=Hormonal 0.65                                 
##                                       TAAR5exp=Negative, treatment=Hormonal
## TAAR5exp=Negative, treatment=Hormonal -                                    
## TAAR5exp=Positive, treatment=Chemo    0.72                                 
## TAAR5exp=Positive, treatment=Hormonal 1.00                                 
##                                       TAAR5exp=Positive, treatment=Chemo   
## TAAR5exp=Negative, treatment=Hormonal -                                    
## TAAR5exp=Positive, treatment=Chemo    -                                    
## TAAR5exp=Positive, treatment=Hormonal 1.00                                 
## 
## P value adjustment method: BH
```

```
## 
##  Pairwise comparisons using Log-Rank test 
## 
## data:  KM and TAAR8exp + treatment 
## 
##                                       TAAR8exp=Negative, treatment=Chemo   
## TAAR8exp=Negative, treatment=Hormonal 0.41                                 
## TAAR8exp=Positive, treatment=Chemo    0.82                                 
## TAAR8exp=Positive, treatment=Hormonal 0.41                                 
##                                       TAAR8exp=Negative, treatment=Hormonal
## TAAR8exp=Negative, treatment=Hormonal -                                    
## TAAR8exp=Positive, treatment=Chemo    0.41                                 
## TAAR8exp=Positive, treatment=Hormonal 1.00                                 
##                                       TAAR8exp=Positive, treatment=Chemo   
## TAAR8exp=Negative, treatment=Hormonal -                                    
## TAAR8exp=Positive, treatment=Chemo    -                                    
## TAAR8exp=Positive, treatment=Hormonal 0.41                                 
## 
## P value adjustment method: BH
```

```
## 
##  Pairwise comparisons using Log-Rank test 
## 
## data:  KM and TAAR9exp + treatment 
## 
##                                       TAAR9exp=Negative, treatment=Chemo   
## TAAR9exp=Negative, treatment=Hormonal 0.85                                 
## TAAR9exp=Positive, treatment=Chemo    0.85                                 
## TAAR9exp=Positive, treatment=Hormonal 0.21                                 
##                                       TAAR9exp=Negative, treatment=Hormonal
## TAAR9exp=Negative, treatment=Hormonal -                                    
## TAAR9exp=Positive, treatment=Chemo    0.85                                 
## TAAR9exp=Positive, treatment=Hormonal 1.00                                 
##                                       TAAR9exp=Positive, treatment=Chemo   
## TAAR9exp=Negative, treatment=Hormonal -                                    
## TAAR9exp=Positive, treatment=Chemo    -                                    
## TAAR9exp=Positive, treatment=Hormonal 0.13                                 
## 
## P value adjustment method: BH
```

##### **Association of TAARs expression and metastases-free survival**

```
## 
##  Pairwise comparisons using Log-Rank test 
## 
## data:  KM and TAAR1exp + treatment 
## 
##                                       TAAR1exp=Negative, treatment=Chemo   
## TAAR1exp=Negative, treatment=Hormonal 0.027                                
## TAAR1exp=Positive, treatment=Chemo    0.827                                
## TAAR1exp=Positive, treatment=Hormonal 0.102                                
##                                       TAAR1exp=Negative, treatment=Hormonal
## TAAR1exp=Negative, treatment=Hormonal -                                    
## TAAR1exp=Positive, treatment=Chemo    0.027                                
## TAAR1exp=Positive, treatment=Hormonal 0.827                                
##                                       TAAR1exp=Positive, treatment=Chemo   
## TAAR1exp=Negative, treatment=Hormonal -                                    
## TAAR1exp=Positive, treatment=Chemo    -                                    
## TAAR1exp=Positive, treatment=Hormonal 0.071                                
## 
## P value adjustment method: BH
```

```
## 
##  Pairwise comparisons using Log-Rank test 
## 
## data:  KM and TAAR2exp + treatment 
## 
##                                       TAAR2exp=Negative, treatment=Chemo   
## TAAR2exp=Negative, treatment=Hormonal 0.187                                
## TAAR2exp=Positive, treatment=Chemo    0.356                                
## TAAR2exp=Positive, treatment=Hormonal 0.028                                
##                                       TAAR2exp=Negative, treatment=Hormonal
## TAAR2exp=Negative, treatment=Hormonal -                                    
## TAAR2exp=Positive, treatment=Chemo    0.141                                
## TAAR2exp=Positive, treatment=Hormonal 0.187                                
##                                       TAAR2exp=Positive, treatment=Chemo   
## TAAR2exp=Negative, treatment=Hormonal -                                    
## TAAR2exp=Positive, treatment=Chemo    -                                    
## TAAR2exp=Positive, treatment=Hormonal 0.018                                
## 
## P value adjustment method: BH
```

```
## 
##  Pairwise comparisons using Log-Rank test 
## 
## data:  KM and TAAR5exp + treatment 
## 
##                                       TAAR5exp=Negative, treatment=Chemo   
## TAAR5exp=Negative, treatment=Hormonal 0.0051                               
## TAAR5exp=Positive, treatment=Chemo    0.5385                               
## TAAR5exp=Positive, treatment=Hormonal 0.5385                               
##                                       TAAR5exp=Negative, treatment=Hormonal
## TAAR5exp=Negative, treatment=Hormonal -                                    
## TAAR5exp=Positive, treatment=Chemo    0.0051                               
## TAAR5exp=Positive, treatment=Hormonal 0.5385                               
##                                       TAAR5exp=Positive, treatment=Chemo   
## TAAR5exp=Negative, treatment=Hormonal -                                    
## TAAR5exp=Positive, treatment=Chemo    -                                    
## TAAR5exp=Positive, treatment=Hormonal 0.5385                               
## 
## P value adjustment method: BH
```

```
## 
##  Pairwise comparisons using Log-Rank test 
## 
## data:  KM and TAAR8exp + treatment 
## 
##                                       TAAR8exp=Negative, treatment=Chemo   
## TAAR8exp=Negative, treatment=Hormonal 0.019                                
## TAAR8exp=Positive, treatment=Chemo    0.917                                
## TAAR8exp=Positive, treatment=Hormonal 0.236                                
##                                       TAAR8exp=Negative, treatment=Hormonal
## TAAR8exp=Negative, treatment=Hormonal -                                    
## TAAR8exp=Positive, treatment=Chemo    0.024                                
## TAAR8exp=Positive, treatment=Hormonal 0.917                                
##                                       TAAR8exp=Positive, treatment=Chemo   
## TAAR8exp=Negative, treatment=Hormonal -                                    
## TAAR8exp=Positive, treatment=Chemo    -                                    
## TAAR8exp=Positive, treatment=Hormonal 0.293                                
## 
## P value adjustment method: BH
```

```
## 
##  Pairwise comparisons using Log-Rank test 
## 
## data:  KM and TAAR9exp + treatment 
## 
##                                       TAAR9exp=Negative, treatment=Chemo   
## TAAR9exp=Negative, treatment=Hormonal 0.244                                
## TAAR9exp=Positive, treatment=Chemo    0.853                                
## TAAR9exp=Positive, treatment=Hormonal 0.035                                
##                                       TAAR9exp=Negative, treatment=Hormonal
## TAAR9exp=Negative, treatment=Hormonal -                                    
## TAAR9exp=Positive, treatment=Chemo    0.244                                
## TAAR9exp=Positive, treatment=Hormonal 0.853                                
##                                       TAAR9exp=Positive, treatment=Chemo   
## TAAR9exp=Negative, treatment=Hormonal -                                    
## TAAR9exp=Positive, treatment=Chemo    -                                    
## TAAR9exp=Positive, treatment=Hormonal 0.015                                
## 
## P value adjustment method: BH
```
