# Supplementary material for: The Expression of Trace Amine-Associated Receptors (TAARs) in Breast Cancer Is Coincident with the Expression of Neuroactive Ligand–Receptor Systems and Depends on Tumor Intrinsic Subtype
Source: Biomolecules. 2023 Sep 7;13(9):1361. doi: 10.3390/biom13091361 (PMC10526748; doi:10.3390/biom13091361)
Supplement: Supplementary file 1 [file biomolecules-13-01361-s001.zip › Supplementary S3.html]

Supplementary 3: association of TAARs expression in breast carcinoma disease recurrence after the treatment including neoadjuvant taxane-anthracycline chemotherapy


# Supplementary 3: association of TAARs expression in breast carcinoma disease recurrence after the treatment including neoadjuvant taxane-anthracycline chemotherapy

This supplementary includes the results of the Kaplan-Meier
recurrence-free survival analysis for patients enrolled in the study of
neoadjuvant taxane-anthracycline chemotherapy in breast cancer
(GSE25066). Expression data are represented for TAAR2 and TAAR5 in this
dataset. We applied the threshold log2 expression = 5.0 to classify
tumor samples as TAAR-positive or TAAR-negative for each TAAR gene
expression.

##### **Association of TAARs expression and recurrence-free survival (time in years)**
